# A nutritional profile of patients with tuberculosis at Standerton Tuberculosis Specialised Hospital, Mpumalanga, South Africa

**DOI:** 10.4102/hsag.v26i0.1594

**Published:** 2021-07-26

**Authors:** Janke Wessels, Mariette Nel, Corinna M. Walsh

**Affiliations:** 1 Department of Nutrition and Dietetics, Faculty of Health Sciences, University of the Free State, Bloemfontein, South Africa; 2 Department of Biostatistics, Faculty of Health Sciences, University of the Free State, Bloemfontein, South Africa

**Keywords:** tuberculosis, nutrition, malnutrition, anthropometric measurements, human immunodeficiency virus

## Abstract

**Background:**

Tuberculosis (TB) is strongly influenced by nutritional status, with nutrition interventions being likely to have an impact on the prevalence of disease, response to drugs and quality of life.

**Aim:**

The aim of this research study was to determine the nutritional profile of patients with TB and TB and human immunodeficiency virus (HIV) co-infection.

**Setting:**

The study was conducted at Standerton TB Specialised Hospital, Mpumalanga.

**Methods:**

A cross-sectional study was undertaken. A structured interview was conducted by the researcher with each patient. The Malnutrition Universal Screening Tool (MUST) was used to determine the risk of malnutrition. Weight, height, mid-upper arm circumference (MUAC) and triceps skinfold were measured using standard techniques. Biochemical parameters that were part of the routine hospital procedure were noted.

**Results:**

More than two-thirds of the participants (68%) were found to be HIV positive. Food-related side effects included loss of appetite (59%) and dry mouth (48%). According to the MUST, 70% had a high risk of malnutrition. The median body mass index (BMI) was in the underweight category at 18.3 kg/m². About half of the participants had low MUAC measurements (51%) and triceps skinfold measurements below the 15th percentile (49.9%), indicating malnutrition. Most participants had low albumin and haemoglobin levels (79% and 92%, respectively).

**Conclusions:**

Patients with both TB and TB and HIV co-infection had a compromised nutritional status and an increased risk for developing malnutrition. Interventions aimed at addressing malnutrition could make a meaningful contribution to improving the quality of life in these patients.

**Contribution:**

This research provides evidence on the nutritional profile of patients with tuberculosis at Standerton TB Specialised Hospital, it gives opportunity to extend this research project to confirm these findings in other TB burdened areas.

## Introduction

Tuberculosis (TB) is the leading cause of morbidity and mortality rates, especially in middle- and low-income countries (WHO 2013), with a quarter of the population being infected with TB globally (WHO 2020). Africa alone accounted for 25% of TB cases in 2019, and more specifically, South Africa (listed as a high-burden TB country) accounted for 3.6% of TB cases in 2019 (WHO 2020).

Tuberculosis is strongly influenced by nutritional status (Kant, Gupta & Ahluwalia 2015), with nutrition interventions being likely to have an effect on the prevalence of active disease, response to drug therapy and quality of life (Cegielski, Arab & Cornoni-Huntley 2012).

Adequate nutrition plays a critically important role in supporting the health and quality of life of people with TB and human immunodeficiency virus (HIV) (Kant et al. 2015; Martin & Sabina 2019). Immune function and nutritional status are closely related, and nutrition, immune function and infection interact in complex ways (Martin & Sabina 2019). Malnutrition is a serious global health problem, which is often not adequately addressed in public health programmes (Rudolph et al. 2013).

Malnutrition may predispose to TB and having TB increases one’s risk of developing malnutrition, with HIV co-infection further increasing the risk of malnutrition – HIV, TB and malnutrition result in a vicious cycle, with the one epidemic exacerbating the other (Martin & Sabina 2019). Malnutrition furthermore increases the risk for developing immunodeficiency and infection, with TB infection leading to malabsorption, wasting and poor nutritional status (Kant et al. 2015).

Several researchers have confirmed the link between malnutrition and TB (Kant et al. 2015; Karyadi et al. 2000; Martin & Sabina 2019; Van Lettow et al. 2004; Villamor et al. 2006). Both malnutrition and HIV infection are associated with an increased risk of progression from the latent TB infection to active TB disease because of the negative impact that dietary deficiencies have on the cell-mediated immune response (Kant et al. 2015; Lönnroth et al. 2010; Martin & Sabina 2019). TB (and HIV infection) is often associated with poor dietary intake, nutrient deficiencies, malnutrition and wasting (Kant et al. 2015; Martin & Sabina 2019; WHO 2020).

Considering the negative impact of poor nutritional status on outcomes in patients with TB and TB or HIV co-infection, the purpose of this research study was to determine the nutritional profile (which included demography, experience of food-related side effects, the Malnutrition Universal Screening Tool (MUST) score, anthropometric indicators and biochemical parameters) of patients with TB and TB and HIV co-infection at Standerton TB Specialised Hospital, Mpumalanga, where information of this nature has not been previously collected. This information can contribute to the identification of specific areas that need to be taken into account in nutrition interventions, which may, in turn, play a vital role in improving the outcome and prognosis of patients with TB and TB and HIV co-infection.

## Methods

### Study design

An analytical cross-sectional study was conducted as qualitative information related to the nutritional profile of participants was determined and analysed in the sample of the patients. Numerical data collected from the subgroups of participants using questionnaires were compared by statistical analysis. As groups were compared, the design is not only descriptive but cross-sectional in nature.

### Target population and sampling

A convenience sample of 100 newly admitted hospitalised patients in Standerton TB Specialised Hospital, Mpumalanga, were included, after they agreed to participate. The study population included all patients between 20 and 65 years with TB and TB/HIV co-infection who gave informed consent to participate. Only patients with pulmonary TB were included, whilst those with drug-resistant TB were excluded (as these patients are often in isolation). Patients with any additional diagnoses other than TB and TB with HIV co-infection, pregnant or lactating patients, and mentally or physically disabled patients were excluded.

### Methods and techniques

#### Eating-related side effects and Malnutrition Universal Screening Tool

A questionnaire was designed by the researcher to obtain information related to demographic factors and eating-related side effects that are often reported in the literature (loss of appetite, sore mouth, dry mouth, nausea, vomiting, constipation, diarrhoea and night sweats experienced during the past 7 days). This was followed by the existing MUST that determines the overall risk of malnutrition.

The MUST consists of three steps: Step 1 classifies a patient according to body mass index (BMI) (> 20 kg/m² = 0; 18.5 kg/m² – 20 kg/m² = 1; < 18.5 kg/m² = 2). Step 2 evaluates the percentage of unplanned weight loss during the past 3–6 months (< 5% = 0; 5% – 10% = 1; > 10% = 2) based on self-reported weight loss. Step 3 evaluates whether the patient is acutely ill and whether there has been any nutritional intake over the past ≥ 5 days; if so, the patient scores an additional two points. The overall risk of malnutrition is then determined by adding the scores for steps 1–3 together. A low risk of malnutrition equals a total score of 0, a medium risk equals a total score of 1 and a high risk equals a total score of ≥ 2 (Bapen 2003).

The MUST is internationally validated and regarded as an accurate tool to evaluate the risk of malnutrition in adult patients (Bapen 2003; Miyata, Tanaka & Ihaku 2013). Only one person, namely, the trained researcher completed the questionnaires in a personal interview with each participant.

#### Anthropometric measurements

Anthropometric indicators of nutritional status included height, weight, mid-upper arm circumference (MUAC) and triceps skinfold. Body mass index was interpreted according to the World Health Organization (WHO) categories of BMI, with underweight < 18.5 kg/m^2^, normal weight 18.5 kg/m^2^ – 4.9 kg/m^2^ and overweight > 25 kg/m^2^ (WHO 2006). An MUAC of < 22 cm for females and < 23 cm for males indicated malnutrition (Tang et al. 2013). Triceps skinfold measurements were interpreted according to the Triceps Skinfold Norms from NHANES 2003–2006 (Lee & Nieman 2013). Triceps skinfold measurements below the 15th percentile indicated malnutrition (Lee & Nieman 2013).

All anthropometric measurements were measured by standard procedures. Weight was measured with an electronic platform scale (TCS-200-RT) to the nearest 0.1 kg. Height was measured using a stadiometer (TCS-200-RT) with a vertical scale of 2 m and a sliding head-piece to the nearest 0.5 cm (Kirenga et al. 2015). The mid-upper arm circumference was measured using a non-stretch flexible tape-measure, taken three times and an average calculated to the nearest 1 mm. Triceps skinfold was measured using a caliper (Body Care). The measurement of the triceps skinfold was performed at the midpoint of the upper right arm, between the acromion process and the tip of the olecranon, with the arm hanging relaxed. The triceps skinfold measurement was taken three times, and the average calculated to the nearest 2 mm (Lee & Nieman 2013).

The scale was tarred before each measurement. In order to ensure reliability, anthropometric measurements were taken by the same trained researcher (a registered dietitian) according to standard procedures (Lee & Nieman 2013).

#### Biochemical parameters

The blood of participants was drawn as part of routine procedures in the hospital by a professional nurse (total protein, albumin, CD_4_ cell counts, MCV and haemoglobin) and was analysed in an accredited laboratory using standard laboratory techniques (National Health Laboratory Service, Standerton Laboratory, Practice number: 5200296) and established cut-off points.

### Pilot study

A pilot study was conducted on the first five patients who met the inclusion criteria and provided informed consent to determine the feasibility of the methodology. No changes were made to the questionnaire. Data of these participants were thus included in the main study.

### Data collection

Eligible participants signed informed consent in their language of choice (English/IsiZulu) after the purpose and procedure of the project had been explained to them in their home language. Once informed consent had been obtained, participants were interviewed and assessed by the researcher.

### Statistical analysis

Descriptive statistics, namely, frequencies and percentages for categorical data, and medians and percentiles for continuous data were calculated. Associations between variables were calculated and described using the Kruskal–Wallis test for numerical data and using Fisher’s exact test for categorical data. Statistical significance was determined using 95% confidence intervals (CIs) for differences in medians or percentages. All analyses were completed by the Department of Biostatistics at the University of the Free State (UFS) using SAS software.

### Ethical considerations

Ethics approval was obtained from the Provincial Health and Research Ethics Committee (PHREC) of Mpumalanga Department of Health (PHREC MP_2015RP38_556) and the Health Sciences Research Ethics Committee of the University of the Free State (UFS) (ECUFS 56/2015). Informed consent was obtained from the participating patients. Questionnaires and anthropometric measurements were completed and taken with each participant in a structured interview with the researcher. The interviews took place in a private room.

## Results

The sample included 100 participants (60 males and 40 females) with a median age of 39.2 (range 20.3–63.5) years. More than two-thirds (68%) were HIV positive, with HIV co-infection being slightly higher amongst women (70%) than men (66.7%).

### Food-related side effects

The majority of participants had experienced loss of appetite (59%) followed by a dry mouth (48%). Patients with TB and HIV co-infection experienced more food-related side effects than those without HIV co-infection; however, the differences were not significant. About one-third of the participants (29%) experienced two side effects, and almost a quarter (24%) reported experiencing four to seven side effects.

## Malnutrition universal screening tool

The overall risk of malnutrition is summarised in Table 1. More than half (51%) of the participants had a BMI of < 18.5 kg/m², and almost half (48%) of them had experienced more than 10% weight loss during the past 3–6 months. According to the MUST, more than two-thirds (70%) of the participants had a high risk of malnutrition (total score of ≥ 2). Almost a quarter (22%) of participants had a medium risk of malnutrition (total score of 1), and only 8% had a low risk of malnutrition (total score of 0).

**TABLE 1:** Overall risk of malnutrition.

*n* = 100	Frequency	Cumulative frequency
**BMI score**		
0 (> 20 kg/m²)	36	36
1 (18.5 kg/m² – 20 kg/m²)	13	49
2 (< 18.5 kg/m²)	51	100
**Weight loss score (unplanned weight loss in the past 3–6 months)**		
0 (< 5%)	38	38
1 (5% – 10%)	14	52
2 (> 10%)	48	100
**Acute disease score (looking at food-related side effects)**		
0	82	82
2 (has been or is likely to be no nutritional intake for > 5 days)	18	100
**Total score (BMI score + Weight loss score + Acute disease score)**		
0	22	22
1	8	30
2	15	45
3	15	60
4	29	89
5	4	93
6	7	100
**Overall risk for malnutrition**		
High (score of ≥ 2)	70	70
Medium (score of 1)	22	92
Low (score of 0)	8	100

BMI, body mass index.

### Anthropometric indicators

The median weight lost (self-reported) during the last 3–6 months was 6 kg (2 kg – 14 kg). Compared with patients with only TB, a significantly more number of patients with TB and HIV co-infection had experienced an unplanned weight loss of > 10% during the past 3–6 months (Table 2).

**TABLE 2:** Unplanned weight loss; comparing tuberculosis with and without human immunodeficiency virus co-infection.

Variable	TB with HIV co-infection (*n* = 68)		TB without HIV co-infection (*n* = 31)		95% CI for the percentage difference
	*n*	%	*n*	%	
Unplanned weight loss during the past 3–6 months	57	83.82	20	64.52	1.5; 38.2*
**Percentage of unplanned weight loss**					
< 5%	11	19.30	5	25.00	-
5–10%	6	10.53	7	35.00	-
> 10%	40	70.18	8	40.00	-
Compared > 10%	-	-	-	-	5.3; 51.0*

TB, tuberculosis; HIV, human immunodeficiency virus; CI, confidence intervals.

* , Statistically significant difference.

Body mass index, MUAC and triceps skinfold measurements are presented in Table 3 for median values and in Table 4 for categorical values. Median BMI was 18.3 kg/m² (men: 18.2 kg/m²; women: 20.6 kg/m²), with more than half (53%) of the participants having a BMI of < 18.5 kg/m². Male participants had a median BMI in either the underweight or normal range. The BMI of women ranged from underweight to obese, with 30% of women having BMI scores above the normal range. The median MUAC of participants was 22.6 cm (men: 22.5 cm; women: 24.2 cm). Based on MUAC, more than half (51%) of the participants were classified as malnourished. Nearly half of the participants (49.9%) had triceps skinfold measurements below the 15th percentile (40.8% in the 5th percentile and 9.1% in the 10th percentile), which indicates malnutrition. Men reported significantly lower median triceps skinfold measurements than women (men: 13.0 mm; women: 19.5 mm), 95%CI [–10; –2].

**TABLE 3:** Body mass index, mid-upper arm circumference and triceps skinfold (median), comparing genders using 95% confidence intervals.

Anthropometric indication	Total group		Men (*n* = 60)		Women (*n* = 40)		95% CI for median difference
	Median	Range	Median	Range	Median	Range	
BMI (kg/m²)	18.3	11.9–41.7	18.2	13.0–23.9	20.6	12.2–41.7	−4.7; 0.0
MUAC (cm)	22.6	14.1–42.7	22.5	16.4–29.5	24.2	14.1–42.7	−4.0; 0.4
Triceps skinfold (mm)	14.0	5.0–38.0	13.0	5.0–28.0	19.5	5.0–38.0	−10.0; -2.0*

CI, confidence intervals; BMI, Body mass index; MUAC, mid-upper arm circumference.

* , Statistically significant difference.

**TABLE 4:** Body mass index, mid-upper arm circumference and triceps skinfold (categories).

Measurement	*n* = 100	Men (*n* = 60)	Women (*n* = 40)
**BMI (%)**			
< 18.5: Underweight	53.0	65.7	47.5
18.5–24.9: Normal	35.0	43.3	22.5
25.0–29.9: Overweight	7.0	0.0	17.5
> 29.9: Obese	5.0	0.0	12.5
**MUAC (%)**			
Malnourished†	51.0	41.7	60.0
Normal‡	49.0	58.3	40.0
**Triceps skinfold (%)**			
5th percentile	40.8	8.3	32.5
10th percentile	9.1	6.6	2.5
15th percentile	7.5	5.0	2.5
25th percentile	22.6	10.0	12.5
50th percentile	34.1	21.6	12.5
75th percentile	56.6	36.6	20.0
85th percentile	15.8	8.3	7.5
90th percentile	9.1	1.6	7.5
95th percentile	4.1	1.6	2.5

BMI, Body mass index; MUAC, mid-upper arm circumference.

† , MUAC malnourished: men (< 23 cm) and women (< 22 cm).

‡ , MUAC normal: men (≥ 23 cm) and women (≥ 22 cm).

### Biochemical parameters

Biochemical parameters of participants are illustrated in Table 5 for median values and Table 6 for categorical values. The majority (61%) of participants had total protein values in the normal range, with 11% below normal and 28% above normal. The median value for total protein was 73.1 g/L, which falls within the normal range of 60 g/L – 78 g/L. Nearly 80% of participants had albumin values below the normal range of 35 g/L, and the median albumin value was 29.0 g/L. Lower albumin levels were significantly more present in males than in females (95%CI [2.7%; 35.9%]) (men: 86.7%; women: 67.5%). The median CD_4_ cell count was 179 mm³, which is far below the lower normal cut-off value of 500 mm³. More than 80% (83.3%) of participants had a CD_4_ cell count below 500 mm³, and 57% had a CD_4_ cell count below 350 mm³. Forty percent had MCV within the normal range of 79.1 f/L – 89.0 f/L, and 43% had an MCV above the normal range, whilst 17% of participants had an MCV below the normal range. The median haemoglobin level was 10.5 g/dL, which is below the lower normal range for males and females. The majority of participants (92%) had haemoglobin levels below the normal range. Males reported significantly lower haemoglobin levels than females (men: 96.7%; women: 85.0%), 95%CI [0.4%; 25.9%].

**TABLE 5:** Biochemical parameters (median) according to gender.

Measurement	*n*	Median	Range	Men		Women		95%CI for median difference
				Median	Range	Median	Range	
Total protein (g/L)	100	73.10	46.0–104.0	71.5	46.0–104.0	74.7	48.0–96.0	−9; 1.0
Albumin (g/L)	100	29.00	14.0–49.0	29.0	14.0–47.0	29.0	18.0–49.0	−5; 1.0
CD4 cell count (mm³)	76	179.00	4.0–995.0	234.0	4.0–737.0	174.0	8.0–995.0	−116; 59
MCV (f/L)	100	88.35	69.8–112.6	88.4	71.6–112.6	88.4	69.8–100.7	−3.2; 3.5
Haemoglobin (g/dL)	100	10.50	6.3–16.0	10.9	6.3–16.0	10.2	8.0–15.6	−0.4; 1.2

CI, confidence intervals; MCV, mean corpuscular volume.

**TABLE 6:** Biochemical parameters (categories) according to gender.

Measurement	Men (*n* = 60)	Women (*n* = 40)	95% CI for the percentage difference
**Total protein (%) (*n* = 100)**	-	-	−12.7; 13.8
< 60 g/l: Low	11.7	10.0	
60–78 g/l: Normal	63.3	57.5	
> 78 g/l: High	25.0	32.5	
**Albumin (%) (*n* = 100)**	-	-	2.7; 35.9*
< 35 g/l: Low	86.7	67.5	
35–52 g/l: Normal	13.3	32.5	
> 4.9 mg/l: High	100	100	
**CD_4_ cell count (%) (*n* = 76)**	-	-	−8.6; 26.9
> 500 mm³: Low	87.2	79.3	
500–2000 mm³: Normal	12.8	20.7	
**MCV (%) (*n* = 100)**	-	-	−17.0; 13.5
< 79.1 f/l: Low	16.7	17.5	
79.1–89.0 f/l: Normal	40.0	40.0	
> 89.0 f/l: High	43.3	42.5	
**Haemoglobin (%) (*n* = 100)**	-	-	0.4; 25.9*
Lower than normal range	-	-	
< 14.3 g/dl	96.7	-	
< 12.1 g/dl	-	85.0	
Between normal range	-	-	
14.3–18.3 g/dl	3.3	-	
12.1–16.3 g/dl	-	15.0	

CI, confidence intervals; MCV, mean corpuscular volume.

* , Significant difference.

## Discussion

The aim of this study was to describe the nutritional profile of patients with TB. The sample was characterised by a high overall risk for malnutrition (70%). The median BMI was in the underweight category at 18.3 kg/m^2^, and about half of participants had low MUAC and triceps skinfold measurements. Most participants had low albumin and haemoglobin values.

Food-related side effects experienced by patients with TB and TB/HIV co-infection may be caused by the disease, the treatment, or a combination of the two. Several food-related side effects have been reported to be related to the use of TB medications (Isoniazid and Rifampicin) (Kant et al. 2015; SADoH 2014; WHO 2020), especially in those with TB and HIV co-infection (Martin & Sabina 2019). Although a higher percentage of patients with TB and HIV co-infection in the current study experienced more food-related side effects than those without HIV, the difference was not significant.

The side effects that were experienced the most include loss of appetite and dry mouth. Reasons for this may be because of changes in appetite regulatory hormones, malnutrition or possibly an unidentified risk factor for TB (Chang et al. 2013). Commonly reported side effects in patients with TB in Uganda, Iran and Pakistan included loss of appetite, loss of weight, dyspnoea, fatigue, weakness, fever, night sweats, chest pain, and haemoptesia (Kirenga et al. 2015; Nezhad et al. 2012; Shaikh et al. 2012). Nezhad et al. (2012) concluded that as the number of reported side effects increased, the total recovery time also increased.

Although it is well recognised that there is a link between TB and malnutrition, the precise mechanisms that are involved are unclear (Lombardo, Swart & Visser 2012; Martin & Sabina 2019). A study amongst patients with TB conducted by Boloor, Amina and Padubidri (2012) in India reported similar levels of malnutrition as those identified in the current study using the Mini Nutritional Assessment Short-Form (MNA-SF).

Involuntary weight loss, wasting and cachexia are common findings reported in patients with TB. All of the above processes are most likely the result of a combination of factors, including increased nutrient losses, altered metabolism, and decreased appetite and food intake, which are all directly linked to a poor prognosis (Chang et al. 2013; Kirenga et al. 2015; Nezhad et al. 2012). The current study also found involuntary weight loss to be very common in patients with TB, especially in those with TB and HIV co-infection.

According to the WHO, low BMI (< 18.5 kg/m²) is the best predictor of weight-related morbidity (WHO 2013). Several cross-sectional studies have confirmed a lower BMI in adults with TB disease, together with an increased risk for mortality and micronutrient deficiencies (Lombardo et al. 2012; Musuenge, Poda & Chen 2020; Van Lettow, Fawzi & Semba 2003). According to Hanrahan et al. (2010), BMI may be a useful surrogate marker of TB risk or mortality in HIV-positive individuals.

Rudolph et al. (2013) reported that the average BMI in adult South African males and females with TB from Alexandra, Johannesburg was 19.2 kg/m² (the lower range of normal) and 23.3 kg/m² (normal), respectively. Even though the BMI of South African females with TB were within the normal range, it was still lower than that of the general population (where most women are overweight and obese). The BMI scores in the current study were even lower than the mean BMI reported by Rudolph et al. (2013) at 18.2 kg/m² for males and 20.6 kg/m² for females.

In the current study, more than half of the participants fell in the low BMI category (< 18.5 kg/m²). Boloor et al. (2012) reported even higher levels of underweight in hospitalised patients with TB in India, where 79% had a BMI score of ≤ 18.0 kg/m. Persons with a BMI in the underweight category have an increased risk for developing TB (odds ratio: 2.6) (Horsburgh, Von Ryan & Baron 2012). Cegielski et al. (2012) reported an even higher risk of 5.5–12.5 for TB in persons with a low BMI, little subcutaneous fat or low skeletal muscle. In the current study, a small percentage of women had BMI scores in the overweight and obese category at 17.5% and 12.5%, respectively. More research related to the relationship between very low and very high BMI levels and TB is needed (Hanrahan 2010; Lönnroth et al. 2012).

Patients with a low BMI also tend to have a low MUAC (UNAIDS 2014). Boloor et al. (2012) and Karyadi et al. (2000) have also reported significantly higher proportions of patients with a very low MUAC (< 20 cm) amongst patients with TB from Indonesia and India compared with the general population. Lombardo et al. (2012) reported that patients newly diagnosed with TB had median BMI and MUAC values at the lower end of the normal ranges at 18.8 kg/m² and 23.4 cm, respectively. In the current study, patients with TB and TB and HIV co-infection had median MUAC values at the lower end of normal (22.6 cm), and 51% fell in the low MUAC category, indicating malnutrition.

Low skinfold measurements are commonly found in patients with TB (Kant et al. 2015), indicating a low lean body mass. In the current study, nearly half of the participants reported triceps skinfold measurements below the 15th percentile, and a significantly more number of men had a lower triceps skinfold thickness than women. Compared with controls, low triceps skinfolds amongst patients with TB have been confirmed in studies from the United States and Indonesia (Cegielski et al. 2012; Karyadi et al. 2000).

The MUST assessment is based on self-reported weight loss and loss of appetite, whereas BMI, MUAC and triceps skinfold measurements are objective measurements of malnutrition. Nevertheless, both self-reported and objective measurements of malnutrition indicated that participants in the current study were either at a high risk of malnutrition or already malnourished at admission.

Serum protein concentrations can be useful for assessing protein status to evaluate the response to nutritional support and to determine whether a patient is at risk of experiencing medical complications, on condition that there is no acute phase response present because of metabolic stress (which is likely in patients with TB) (Lee & Nieman 2013). In the current study, the majority of patients had serum protein levels in the normal range, and almost a third of patients had serum protein levels above the normal range. Total protein is often elevated in patients infected with HIV because of a condition termed ‘polyclonal gammapathy’ (Alexianu & Dan 2009), which may have been the case in some of the participants in the current study. With metabolic stress (such as TB and HIV) the albumin fraction decreases but the globulin fraction increases (high levels of IgA and IgG); thus, the total protein might increase, decrease or be normal, which further explains the results of the current study (Shingdang et al. 2016).

In the current study, a high percentage of participants had an albumin level below the normal cut-off point, with a median albumin value of 29 g/L. Men had significantly lower albumin levels than women. Similar results were confirmed in a study conducted in Singapore amongst patients with TB and HIV co-infection, where a mean albumin value of 29.6 g/ dL was reported (Paton et al. 2003). Serum albumin is an indicator of depleted protein status and decreased protein intake (Lee & Nieman 2013). Hypoalbuminaemia is an important marker of severe malnutrition (Matos & Moreira Lemos 2006) but is not a reliable indicator of nutritional status when an infection is present as albumin is a negative acute phase protein (Mugusi et al. 2003). Low serum albumin levels are strongly associated with an increased risk of TB. This was confirmed in a study amongst adult patients in the United States (Cegielski et al. 2012). Serum albumin concentrations might be a useful diagnostic and prognostic marker for TB in HIV-infected patients (Alvarez-Uria et al. 2013). A study of hospitalised patients with TB in Brazil found that the group of patients who died during hospitalisation had significantly lower mean albumin levels compared with the group of those who survived (26 g/L vs. 31 g/L) (Matos & Moreira Lemos 2006). Alvarez-Uria et al. (2013) reported that a serum albumin level of > 38 g/L was a negative predictor for TB even in settings with a high prevalence, whereas a serum albumin level of < 32 g/L was associated with 85% TB specificity. Thus, correcting a low serum albumin value in the hospital setting through appropriate nutrition intervention is likely to improve the prognosis of patients with TB.

Low haemoglobin levels were present in the majority of participants in the current study and were significantly more noticeable in men, with the reason being unknown. A low MCV (an indication of an iron deficiency) were present in almost a fifth (17%) of participants. A number of studies in the United States, Indonesia, India, Gambia and Tanzania have confirmed that anaemia is particularly common in patients with TB (Boloor et al. 2012; Cegielski et al. 2012; Isanaka et al. 2012; Karyadi et al. 2000; Minchella et al. 2015) predominantly because of anaemia of inflammation (also known as anaemia of chronic disease) (Minchella et al. 2015), which may have been the case in the current study, where this phenomenon was frequently reported. Some studies have reported a lower prevalence of iron deficiency in patients with TB compared with control groups (Feleke, Feleke & Biadglegne 2019; Friis et al. 2009; Van Lettow et al. 2005). The incidence of an iron deficiency in patients with TB is most likely to vary across populations because of contextual factors, such as dietary intake and prevalence of other infections (Isanaka et al. 2012).

We acknowledge that the results of this single-centre study may not be generalised to all patients with TB, and TB and HIV co-infection. Although information related to weight loss and appetite was self-reported, the MUST has been validated as a reliable tool for assessment of nutritional risk in patients with TB (Miyata et al. 2013). This study is well-controlled and, to our knowledge, is the first study to use the MUST to investigate the nutritional profile of hospitalised patients with TB and TB and HIV co-infection in South Africa.

## Conclusion and recommendations

The current study highlighted that patients with TB and TB and HIV co-infection had a poor nutritional status when considering specific food-related side effects, anthropometric measurements and biochemical parameters. These factors elevate their risk of developing malnutrition, as confirmed by the MUST screening tool, which revealed that most were malnourished.

Nutrition interventions cannot replace the medical management of TB, just as medical management of TB cannot replace adequate nutrition. Nutritional screening with validated malnutrition screening tools is, however, seldom performed in public sector hospitals in South Africa, and underlying malnutrition may not be identified at admission. In most cases, the nutritional status of patients is assessed using limited parameters (for instance, clinical judgement, current weight or self-reported weight loss). The findings of the current study provide evidence for recommendations to apply a comprehensive protocol for the nutritional screening and management of patients with TB in order to identify patients at increased risk of mortality and to provide adequate nutritional support.

Furthermore, provisioning of nutritional support to families and contacts of persons with TB is found to prevent the progression of the latent TB disease to active disease.
